# Genetic and functional analysis of HIV-1 Rev Responsive Element (RRE) sequences from North-India

**DOI:** 10.1186/1742-6405-7-28

**Published:** 2010-08-03

**Authors:** Yogeshwar Sharma, Ujjwal Neogi, Vikas Sood, Snigdha Banerjee, Subodh Samrat, Ajay Wanchu, Surjit Singh, Akhil C Banerjea

**Affiliations:** 1 Division of Virology, National Institute of Immunology, JNU Campus, Aruna Asaf Ali Marg, New Delhi-110067, India; 2 Department of Internal Medicine, Post Graduate Institute of Medical Education & Research, Chandigarh, India; 3 Department of Pediatrics, Post Graduate Institute of Medical Education & Research, Chandigarh, India

## Abstract

HIV-1 Rev protein regulates the expression of HIV-1 transcripts by binding to a highly structured stem loop structure called the Rev Responsive Element (RRE) present in the genomic and partially spliced RNAs. Genetic variation in this structure is likely to affect binding of Rev protein and ultimately overall gene expression and replication. We characterized RRE sequences from 13 HIV-1 infected individuals from North India which also included two mother-child pairs following vertical transmission. We observed high degree of conservation of sequences, including the 9-nt (CACUAUGGG) long sequence in stem-loop B, required for efficient binding of Rev protein. All of our 13 RRE sequences possessed G to A (position 66) mutation located in the critical branched-stem-loop B which is not present in consensus C or B sequence. We derived a consensus RRE structure which showed interesting changes in the stem-loop structures including the stem-loop B. Mother-Child RRE sequences showed conservation of unique polymorphisms as well as some new mutations in child RRE sequences. Despite these changes, the ability to form multiple essential stem-loop structures required for Rev binding was conserved. RRE RNA derived from one of the samples, VT5, retained the ability to bind Rev protein under *in vitro *conditions although it showed alternate secondary structure. This is the first study from India describing the structural and possible functional implications due to very unique RRE sequence heterogeneity and its possible role in vertical transmission and gene expression.

## Introduction

HIV-1 displays very high genetic diversity and has been classified into various subtypes and recombinant forms. While subtype B predominates in US and UK, it is subtype C that is predominant in India, China and South Africa. Most of the changes are observed in the Envelope region but other region like p24-Gag is relatively conserved among subtypes and has been exploited to develop ELISA for diagnostic purposes. HIV-1 exploits the splicing machinery very efficiently by using the Rev protein which binds with high affinity and specificity to highly structured cis-acting RNA element present within the coding region of HIV-1 Envelope gene [1] called Rev Responsive Element (RRE). This RRE element folds into 4 well defined stem-loop structures (A to D) and stem-loop B (stem-bulge-stem structure) is critically important for efficient binding with Rev Protein [2]. Natural variations in the RRE sequences can potentially impact on the secondary structure which might modulate the efficiency of Rev binding. Several studies have earlier suggested that the major Rev protein binding site resides in the predicted second branched stem-loop region [1,3] and other regions of the full-length RRE may influence the binding of Rev protein [4]. Rev - RRE interaction is crucial for efficient late gene expression and replication and efforts are being made to develop novel antiviral approaches that interfere with this interaction. RevM10, a transdominant negative Rev protein, was earlier shown to interfere with HIV-1 replication in T-cell lines and also in primary T-cells [5]. RRE element has been exploited as decoy for specific targeting of HIV-1 gene expression and replication [6].

RRE variants are produced when cells are treated with this protein [7]. Very recently resistant mutants were identified due to altered RRE structures in presence of RevM10 protein [8]. These two studies strongly suggest that sequences in RRE can change under pressure that can have great functional implications. Earlier, Ramakrishnan & Ahmad, 2007 [9] carried out genetic and structural studies of the RRE sequences among mother-child pairs from USA where subtype-B is predominant. The extent and nature of genetic variation and its implication on the secondary structures on its known functions in India is lacking where the epidemic is largely driven by subtype C. Studying sequence variation in the mother-infant pairs will provide insights into the evolution and selection pressures exerted.

The interaction of HIV-1 Rev with RRE is critical for viral gene expression and replication of the virus. Data from various geographic location and subtypes would help us to develop strategies in combating HIV infection. As per our knowledge there is no data available on HIV-1 subtype C RRE genetic and functional characteristics. In the present study, we present in-depth genetic and functional analysis of RRE sequences from a cohort of 13 HIV-1 infected individuals from North-India. The sequences were compared with the Indian consensus C and consensus B along with earlier published subtype C RRE sequences from India. A unique region specific conservation along with Subtype C and B specific mutations were observed in all of the stem-loop structures. We further show that RRE sequences derived from one of the samples (VT5) retained the ability to bind to Rev protein under *in vitro *conditions, though the *in silico *analysis detects an alternate secondary structure. This study is first of its kind to characterize HIV-1 subtype C RRE sequences both genetically and functionally.

## Methods

### Patient description

Detailed sequence analysis was carried out from HIV-1 infected individuals from Chandigarh-Punjab region as described in our recent HIV-1 LTR related paper [10]. They were monitored at Post Graduate Institute of Medical Education and Research (PGIMER), Chandigarh by Dr A Wanchu (Clinician and one of the authors) after obtaining all requisite ethical clearances. The clinical features of all the 13 HIV-1 infected individuals are shown in table 1.

**Table 1:** Demographic, clinical parameters of HIV-1 infected individuals.

Subject	Age (in years)	Sex	Mode of Transmission	Time Since Detection	ART Status	CD4 Count during blood collection
NII-PGI-IND-S1	33	M	Heterosexual	5 years	Not on ART	364

NII-PGI-IND-S2	37	M	Heterosexual	-	-	NA

NII-PGI-IND-S3	35	F	Heterosexual	6 years	Last 4 years	253

NII-PGI-IND-S4	23	F	Heterosexual	1 year	Not on ART	NA

NII-PGI-IND-S5	29	M	Heterosexual	1 year	Since 8/2/08	111

NII-PGI-IND-S6	30	M	Heterosexual	1 year	Not on ART	345

NII-PGI-IND-VT1	24	F	Heterosexual	2 years	Last 6 Month	152

NII-PGI-IND-VT2	4	M	Vertical	2 years	Last 6 Month	727

NII-PGI-IND-VT3	30	F	Heterosexual	1 year	Not on ART	233

NII-PGI-IND-VT5	38	F	Heterosexual	3 years	-	96

NII-PGI-IND-VT6	6	M	Vertical	3 years	-	1048

NII-PGI-IND-D1	30	F	Heterosexual	1 years	Not on ART	419

NII-PGI-IND-E3	9	M	Vertical	1 year	Not on ART	NA

N.B. - "indicates unknown, NA- Not available".

### Genomic DNA isolation and analysis of RRE secondary structures

The genomic DNA was isolated from peripheral blood lymphocytes as described by us earlier [11] and subjected to polymerase chain reaction with RRE specific primers. 247 nt long RRE genes were amplified using specific primers common for both subtypes B and C and placed them under CMV/T7 promoter of the expression vector pCDNA3.1 (Promega Biotech.) that was digested with *Hind *III and *Bam *H1. Following primers were used:

1. Forward: 5'- GGC aagctt GAGCAGTGGGAATAGGAGCTTTG

2. Reverse: 5'- GGC ggatcc AGGAGCTGTTGATCCTTTAGGTATCT

The sequence information was generated using T7-specific primers. Indian specific Consensus C sequences were created as describes previously [12]. Sequences were compared with Indian Consensus C and consensus B and Indian RRE subtype C sequences downloaded from Los Alamos Database http://www.hiv.lanl.gov/. The secondary structures were obtained using RNAalifold program of Vienna RNA package that uses the Zuker algorithm as recently reported [13]. At least 4 independent clones were analyzed for each sample to rule out *Taq *polymerase mediated mis-incorporation of nucleotides. A consensus RRE secondary structure was created by using the program described by Gruber *et. al*., 2008 (website http://rna.tbi.univie.ac.at) [13]. All the 4 clones derived from a single individual showed complete similarity among them. Mother-child samples were processed separately to avoid potential cross contamination.

### Rev cloning, purification, in vitro synthesis of RRE RNA and EMSA

Towards this end, we amplified Rev B using pNL4-3 [14] and Rev C using 93IN905 [15] genetic clones, as described above and purified it to homogeneity as GST-fusion proteins after placing them in bacterial expression vector (pGEX4T-2, Amersham Bioscience) following the earlier described protocol [16]. Prior to cloning in the bacterial expression vector, both the exons of the Rev genes were precisely fused using the fusion technology described by us recently [17]. We also amplified 247 nt long RRE fragment using specific primers and placed it under CMV/T7 promoter of the expression vector pCDNA3.1 (Promega Biotech.) that was digested with *Hind *III and *Bam *H1. *Hind *III and *Bam *H1 restriction sites were engineered at the beginning of forward and reverse primers respectively (small case) to facilitate cloning in the expression vector as described above. ^32^P labeled RRE RNA was generated using T7 RNA polymerase and fixed amounts of it was incubated with varying amounts of Rev protein and subjected to EMSA as described earlier [18].

## Results and discussion

### Analysis of RRE nucleotide sequences

83 sequences of subtype B (from USA, Japan, Mayanmar, France and Brazil) and 83 sequences of Indian subtype C, were downloaded from Los Almos data base (Accessed on 13^th ^May 2010). The mean intra-species identity of Subtype B RRE sequences was 95.3% (range 90 -100) and Indian subtype C strains was 95.03% (Range 89-100). The identity between consensus B and C (downloaded from Los Alamos Database) was 94%. Thus RRE region is one of the most conserved regions in the genomic RNA between HIV-1 subtypes B and C and with probably other subtypes as well. The analysis of intra-subtype divergence (genetic distance from Indian consensus sequence) and diversity (intra-subtype genetic variability of North Indian isolates) of these strains showed significant difference (0.105 vs. 0.011, p < 0.00011), thus it is tempting to speculate that North Indian HIV-1 subtype C RRE sequences are highly conserved and the phylogenetic analysis showed a monophyletic clade indicating epidemiological linkage of these samples (data not shown).

**Figure 1:**
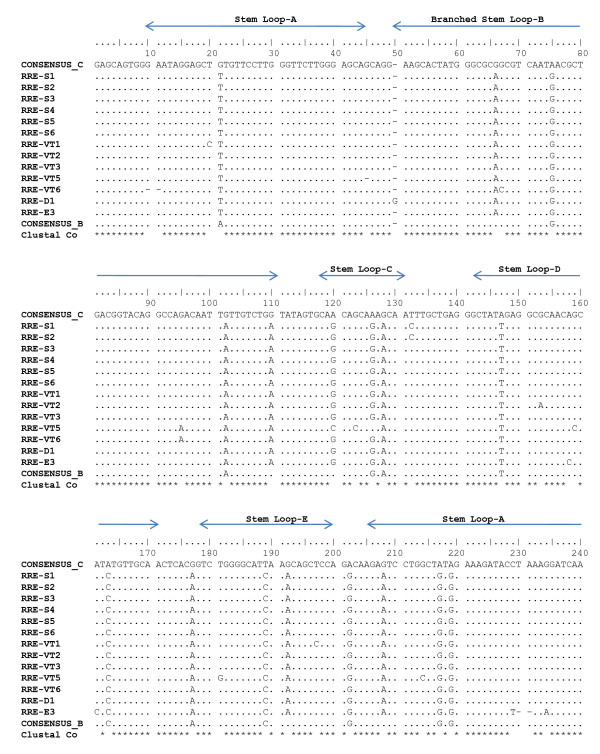
**HIV-1 RRE variants in North India**: HIV-1 RRE sequence analysis and its comparison with a known prototype subtype C (93IN905) [15] and subtype B (pNL4-3) [14]. Five stem loop regions are shown at the top of the sequence. Samples (S1 to E3) were analyzed in this study and compared with other known RRE sequences (with their accession numbers) from India published earlier. Periods indicate similarity and - indicate a deletion. VT1/VT2 and VT5 and VT6 form mother child pairs. Accession numbers FJ649319 to FJ649331 were obtained for all our 13 samples (S1 to E3- sequentially).

When the sequences were compared with the consensus Indian subtype C and consensus B RRE sequences, all the four stem loops (C, D, E and A) showed nucleotide changes that were common with the latter with some unique region specific mutations. In stem loop A, G21A. A208G unique mutation observed in our cohort sequences. It is noteworthy that all of our 13 RRE sequences possessed G66A substitution located in the critical branched-stem-loop B which is neither present in the consensus C nor in Consensus B (Figure 1). In the same region, a unique G110A mutation was observed in North Indian strains. This region is critically involved with the binding of Rev protein. A120G mutation was observed in stem loop C and G192A substitution was in stem loop E.

### Mother- Child transmission of RRE sequences

Two mother child pair samples namely, VT1 (mother) and VT2 (child) and VT5 (mother) and VT6 (child) were analyzed for the evolution or conservation of sequences. All of the stem-loop structures were retained with minor genetic changes that were different in the pair. For example, G123C mutation was observed only in stem-loop C of the mother. On the other hand, G94A unique mutation in stem-loop B was conserved both in mother and child (VT5 & VT6). The critically important 9 nt sequence involved in high affinity binding with Rev protein, was however, completely conserved (figure 1).

### Secondary structure prediction of RRE sequences

**Figure 2:**
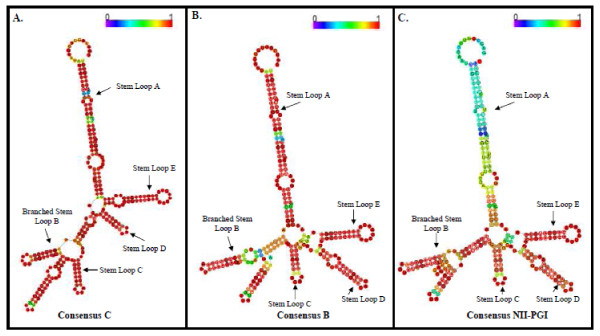
**Predicted consensus secondary RRE structure**: A consensus secondary structure of our RRE sequences were generated from, 20 subtype C (panel A) and B (panel B) and 13 RRE sequences from this study (panel C) which uses multiple sequence alignment program using RNA fold program in the Vienna RNA package (Zuker algorithm) as described in the text. Five (A to E) well defined stem-loop structures including the branched stem-lop B critical for binding Rev protein were identified. In this program the pale colors indicate that a base-pair cannot be formed in some sequences of the alignment.

**Figure 3:**
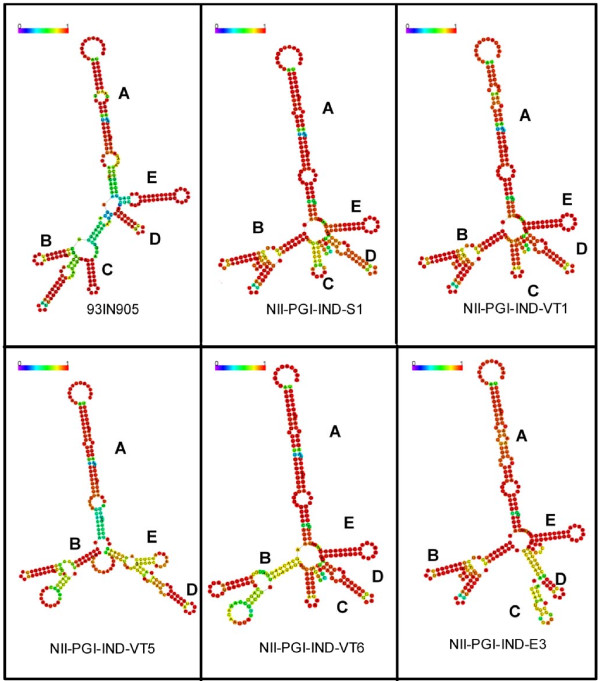
**Secondary structures of RRE variants**: Representative samples were subjected to RNA fold program as described in figure 2. All of these structures display five well defined stem-loop structures (A to E) but show unique changes (described in the text).

A consensus RRE structure was generated using previously published subtype C (figure 2 panel A), subtype B (figure 2 panel B) and all of our 13 RRE sequences (consensus NII-PGI) (figure 2 panel C) and subjected them to multiple sequence alignment program (Vienna RNA conservation coloring). RRE sequence consisted of four stem-loop structures. When the individual RRE sequences were subjected to the RNA folding program, minor variations (in the length or in the size of the minor stem-loops) in the vicinity of well-defined stem-loop structures were observed (figure 3). This secondary structure exhibited an additional stem-loop (as in the case of stem-loop C with E3, a common short stem-loop between stem-loop C and D as in the case of S1 and VT1. Remarkably, gross changes (particularly D and E stem loop structures) in the secondary structures were observed between VT5 (mother) and child VT6 (child) (figure 3).

Our RRE sequence analysis of HIV-1 infected individuals (including mother -child pair samples) suggest that despite heterogeneity, four major stem-loop structures were conserved. This is important for Rev-RRE interaction which governs HIV-1 splicing and replication. Nine nucleotide long sequence (CACUAUGGG) present in stem-loop B was totally conserved in all our 12 samples and also among the early isolates from India (Data not shown). The most important observation was the presence of G to A (66^th ^position) mutation present in the 2^nd ^stem-loop region which was unique to our samples (not observed in Consensus B or C sequences) and argues strongly in favor of selective forces responsible for selection of this mutation. Mutations were observed in other stem-loop regions (A, C, D & E) also. Most of these nucleotide changes are also observed in consensus subtype B RRE sequence. Thus, most of our RRE sequences show similarity with either consensus B or C but the polymorphisms observed show similarity with consensus RRE B sequences. It is tempting to speculate that structural constraints may allow the generation of RRE sequences that are either subtype B or C-specific in this region. It must be pointed out that consensus RRE B and C sequences used here for comparison showed about 94% similarity between each other. Although we have carried out sequence analysis from 4 independent clones, it may still be argued that these mutations are due to mis-incorporation of nucleotides by the Taq polymerase. It is noteworthy that we used high fidelity Taq polymerase (Platinum Taq, Invitrogen). To further rule out this possibility, we isolated HIV-1 genomic RNA from the plasma of HIV-1 infected individuals from two samples (VT5 & VT6) and sequence information generated after PCR matched perfectly with the sequence generated from the DNA clones.

### VT5 RRE binds to Rev B protein efficiently

**Figure 4:**
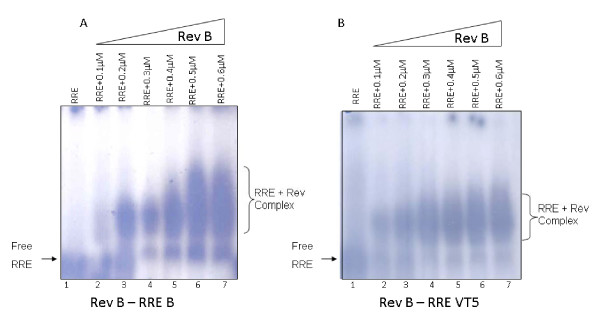
**^32^P labeled RNA was made using T7 RNA polymerase after linearizing the vector as described by the manufacturer (Promega) and as described earlier **[19]. Increasing amounts of purified GST-Rev B or C (0.1 to 0.7 μM) was mixed with fixed amounts of labeled RRE in 10 μl of binding buffer (10 mM HEPES/KOH pH 7.6, 150 mM KCl, 2 mM MgCl_2_, 0.5 mM EGTA, 1 mM DTT, 20% (V/V) glycerol, 3.2 μg E. coli t-RNA) and subjected to EMSA under exactly identical conditions as described before [18].

RRE - B was derived from pNL4-3 [11] and cloned under T7 promoter in pCDNA3.1 (Promega) to generate RRE B RNA. RNA (fixed amounts) and Rev protein (varying amounts) interaction was monitored by EMSA as described earlier [18] and briefly described in the legend to figure # 4. As evident from figure 4, VT5 32P labeled RRE RNA was just as efficient in its ability to interact with Rev protein as RRE- B RNA with Rev protein. We conclude that VT5 derived RRE sequence is functionally relevant and competent though it shows alternate secondary structure. This also suggests that G to A transition (position 66) observed in VT5 RNA did not affect its binding ability to Rev protein. When RRE from VT5 was incubated with Rev C protein (derived from 93IN905), similar observation was made (data not shown).

Majority of HIV-1 infections among infants is due to vertical transmission from mother. It is, therefore, important to characterize various HIV-1 genes with respect to sequence variation or conservation. Our sequence & predicted structural analysis of mother (VT5) and child (VT6) pair indicate that stem-loop D and E have undergone some changes. These kinds of changes in the total number or the length of stem-loop structures in the RRE were reported earlier also [9], in a vertical transmission study carried out between mother and infant pairs. It must be pointed out that the nature of polymorphisms observed in our studies is significantly different than what was observed by Ramakrishnan and Ahmad [9] for subtype-B-specific genes. Despite this kind of heterogeneity, the domains required for Rev protein binding or host protein interaction with RRE was conserved which is crucially important for viral gene expression and replication.

Another remarkable common feature of this study and studies carried out about 9 to 10 years ago [14] was the conserved C to T in stem-loop B, A to G and G to A in stem-loop D and some partially conserved nucleotide changes (G to A) in stem-loop E. Although precise mechanism for this conservation is not known, it is tempting to speculate that certain mutations are uniquely selected in our region. Host factors, besides other factors may potentially influence these changes. This is not surprising because several host factors are known to interact with RRE structures and modulate the splicing ability of Rev protein.

In summary, we genetically characterized the nature of heterogeneity in the RRE sequences from HIV-1 infected individuals from North India along with its impact on the formation of multiple stem-loop structures. These structures show significant differences with respect to either the length or number of stem-loop structures when compared with prototype B and C RRE sequences. Transmission studies with mother-child pair revealed some conserved and new mutations but the ability to form stem-loop structures was retained. RRE derived from one of our samples (VT5) was fully capable of binding the Rev protein with equal efficiency as that of RRE B derived from subtype B (pNL4-3).

How these changes in the secondary structures of RRE RNA affect Rev protein binding in mammalian cells (or host factors), splicing and virus replication may be important for the virus replication.

## Competing interests

The authors declare that they have no competing interests.

## Authors' contributions

YS, UN, VS, SB and SS carried out the experiments. Dr A. Wanchu and Dr S. Singh helped with clinical characterization of the infected samples. ACB is the principal investigator responsible for designing the work and writing the manuscript. All authors read and approved the final manuscript.
